# Epoxy-based multifunctional solid polymer electrolytes for structural batteries and supercapacitors. a short review

**DOI:** 10.3389/fchem.2024.1330655

**Published:** 2024-03-01

**Authors:** Nitai Chandra Adak, Sungmook Lim, Guk-Hwan Lee, Wonoh Lee

**Affiliations:** School of Mechanical Engineering, Chonnam National University, Gwangju, Republic of Korea

**Keywords:** epoxy, solid polymer electrolyte, ionic conductivity, electrochemical property, mechanical strength, thermal stability

## Abstract

The potential applications of epoxy-based solid polymer electrolytes are continually expanding because of their versatile characteristics. These characteristics include mechanical rigidity, nonvolatility, nonflammability, and electrochemical stability. However, it is worth noting that pure epoxy-based solid polymer electrolytes inherently exhibit lower ion transport capabilities when compared to traditional liquid electrolytes. Striking a balance between high mechanical integrity and superior ionic conductivity at room temperature poses a significant challenge. In light of this challenge, this review is dedicated to elucidating the fundamental concepts of epoxy-based solid polymer electrolytes. It will explore various preparation techniques, the incorporation of different nanomaterials into epoxy-based solid polymer electrolytes, and an evaluation of their multifunctional properties. This comprehensive evaluation will cover both mechanical and electrical properties with a specific focus on their potential applications in batteries and structural supercapacitors.

## 1 Introduction

In recent times, the demand for multifunctional structural composite materials has been steadily increasing, primarily fueled by their diverse applications in electric vehicles. This heightened interest has prompted extensive research and development efforts over the past 2 decades, aiming to unlock the potential of multifunctional structural composites as “structural power” storage devices (Snyder et al., 2007a; Carlson et al., 2010). These materials exhibit the capability to bear substantial mechanical loads while simultaneously serving as electrical energy storage devices in the form of batteries, capacitors, and supercapacitors. Despite the promising prospects, the electrolyte component remains a significant challenge in achieving true multifunctionality. Numerous components still require further development and optimization (Asp and Greenhalgh, 2014).

Structural electrolytes, also known as electrolytes for structural energy storage devices, must possess both high ionic conductivity and robust mechanical performance. These requirements present a challenge, as high ionic conductivity typically implies polymer flexibility. While multifunctional materials may not precisely match the specific properties of their single-function counterparts, they contribute to efficiency by consolidating multiple functions into one material or device (Ferreira et al., 2016). Consequently, the combined mass (or volume) of these materials can be reduced. In pursuit of these goals, the literature suggests the ionic conductivity and Young’s modulus targets of 1 m cm^–1^ and 1 GPa, respectively, for structural electrolytes relevant to multifunctional energy storage devices (Shirshova et al., 2013). Currently, electrolytes developed by the reaction-induced phase separation (RIPS) have demonstrated the most promising performance in achieving these goals. In this binary electrolyte system, one phase facilitates ion conduction, while the other imparts mechanical strength and modulus (Ihrner et al., 2017). The most common of this established system is based on vinyl and epoxy formulation. Although this formulation begins as homogeneous, the growth of polymer chains in liquid electrolyte can induce phase separation during the reaction.

**FIGURE 1:**
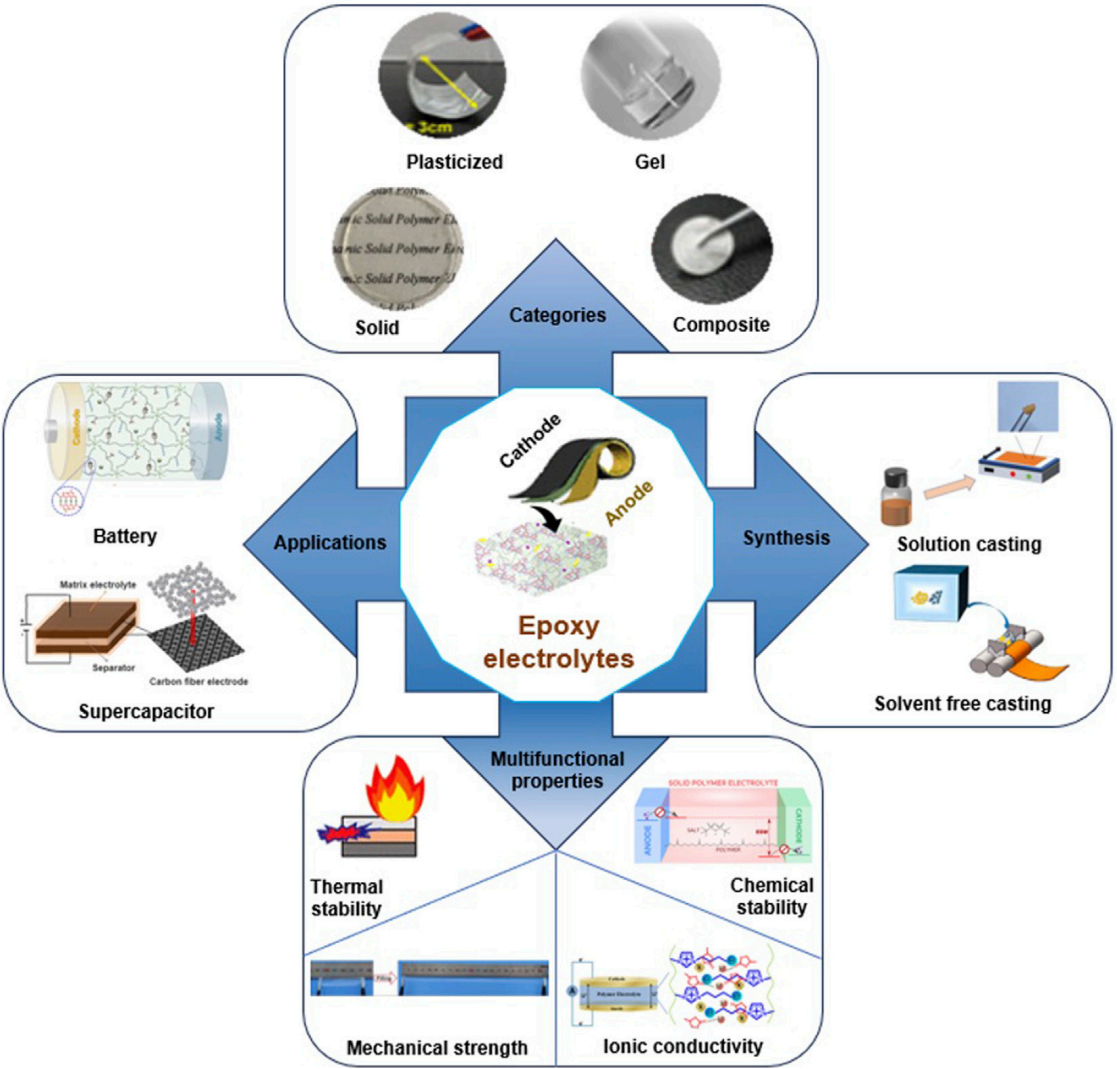
Summary of the multifunctional epoxy-based SPEs for structural energy storage devices. Reprinted with permission from (Qian et al., 2013; Guo et al., 2017; Lu et al., 2017; Baik et al., 2019; Chen et al., 2020; Kato et al., 2020; Marchiori et al., 2020; Song, et al., 2020; Zhou et al., 2020; Han et al., 2022; Zheng et al., 2022; Zhou et al., 2022; Bandyopadhyay et al., 2023) with permission from the American Chemical Society.

As illustrated in Figure 1, epoxy-based solid polymer electrolyte (SPE) systems are highly appealing because of their extensive use in fiber composite systems and their exceptional mechanical, adhesive, thermal, and chemical stability. Researchers have explored various liquid electrolytes to introduce ionic conductivity in these systems, including ionic liquids (ILs), lithium salts, organic solvents, conducting polymers, and nanoparticles, either individually or in combinations (Lim et al., 2019). An alternative promising approach for developing structural electrolytes involves utilizing a two-step RIPS process. This process begins by initiating the reaction in the presence of a sacrificial porogen, followed by its removal and replacement with the final electrolyte. For instance, by filling the porous structure left after removing low molecular weight polyethylene glycol with 1 M lithium hexafluorophosphate (LiPF_6_) in ethylene carbonate/diethyl carbonate, an ionic conductivity of 0.71 m cm^–1^ was achieved (Sakakibara et al., 2017). The Young’s modulus of the electrolyte was significantly enhanced, reaching up to 0.65 GPa, through the incorporation of nano-cellulose, a block copolymer, and an epoxy resin with multiple oxirane rings. However, compared to the one-step RIPS method, this approach is less practical for real-world applications because of its manufacturing complexity. It is noteworthy that the initial composition of the mixture and the curing conditions exert a substantial impact on the properties of structural electrolytes based on the RIPS process.

ILs and their lithium salt solutions have been the focal point of extensive research in the development of epoxy-based structural electrolytes. The resultant materials have exhibited a wide range of combinations of ionic conductivities and mechanical performance, depending on factors such as the epoxy type, curing agent, IL quantity, and the addition of various compounds. In particular, bisphenol A-type diglycidyl ether (DGEBA), a frequently used epoxy resin in structural applications, has garnered significant attention in the context of RIPS-based structural electrolytes, when combined with 1-ethyl-3-methylimidazolium bis(fluorosulfonyl)imide (EMIM-TFSI) (Han et al., 2022). A polymer blend of poly (ethylene glycol) (PEG) and DGEBA epoxy resin have demonstrated improved combination mechanical performance and ionic conductivity (Choi and Jung, 2018).

Given the use of two different crosslinkers, direct comparisons can pose challenges; however, it is reasonable to attribute the reduced performance of the electrolyte containing PEG-DGEBA to the inherent flexibility of PEG compared to the more rigid DGEBA. It is crucial to note that the IL inclusion is not always necessary to create an ion-conducting phase in structural electrolyte formulations based on DGEBA. For instance, lithium trifluoromethanesulfonate (LiTF), a lithium salt, has been employed in conjunction with lower molecular weight PEG (Sundararajan et al., 2018). To counteract the plasticizing effects of PEG, nano-silica particles were added to the epoxy, resulting in a structural electrolyte with a tensile modulus of 135 MPa and an ionic conductivity of 0.086 m cm^–1^ (Feng et al., 2017). While fully formulated commercial multicomponent epoxy resin systems can offer improved performance, pure DGEBA systems remain relatively straightforward to understand and study. For example, a structural electrolyte was formulated using the commercial epoxy resin MTM57 in a solution of 2.3 M lithium bis(trifluoromethanesulfonyl)imide salt (LiTFSI) in EMIM-TFSI. This electrolyte exhibited high ionic conductivity at 0.43 m cm^–1^ and Young’s modulus of 230 MPa (Shirshova et al., 2014a).

Another critical factor influencing the balance between modulus and ionic conductivity is the ratio of liquid electrolyte to resin, often achieved through changes in morphology and phase proportions. When the liquid electrolyte content is low, or only slightly higher than what is soluble in the epoxy, it results in the formation of a microstructure where droplets of the liquid electrolyte are dispersed randomly within the bulk phase of the epoxy. While the mechanical properties of these samples remain high, approaching that of the neat epoxy, the ionic conductivity decreases due to the microstructure formation. However, once a certain limit of liquid electrolyte incorporation in the epoxy resin is reached, a continuous phase is formed. The performance of this homogeneous structural electrolyte can be enhanced by altering the morphology and the final structures created by the epoxy phase (Shirshova et al., 2014b). In this context, morphologies vary widely, ranging from bi-continuous strut-like networks to fused epoxy nodules surrounded by the liquid electrolyte. Among these structures, the most effective combinations tend to use strut-like structures. The characteristic length scale of the microstructure is determined by the relative diffusion and curing kinetics. Therefore, it is crucial for the characteristic length scale of the structural electrolyte to align with the pore size of the electrode material.

So far, research efforts have concentrated on the development of multifunctional structural electrolytes to achieve a balance in the mechanical, thermal, chemical, and electrical properties of bulk polymer electrolytes without altering the electrode materials and architectures. Epoxy-based SPEs are particularly desirable in this context, given their potential applications in structural energy storage devices such as batteries and capacitors. However, to the best of our knowledge, a comprehensive review focusing solely on epoxy-based multifunctional SPEs, considering their mechanical rigidity, nonvolatility, nonflammability, electrochemical stability, and ion transport mechanism, is currently lacking in the literature. Therefore, this review is dedicated to elucidating the fundamental concepts of epoxy-based SPEs. It will cover various preparation techniques, the incorporation of different nanomaterials into epoxy-based solid polymer electrolytes, and an evaluation of their multifunctional properties. This assessment will include both mechanical and electrical properties, with a specific focus on their potential applications in structural batteries and supercapacitors.

## 2 Fundamentals of epoxy-based SPEs

### 2.1 Basic composition and working principle

**FIGURE 2:**
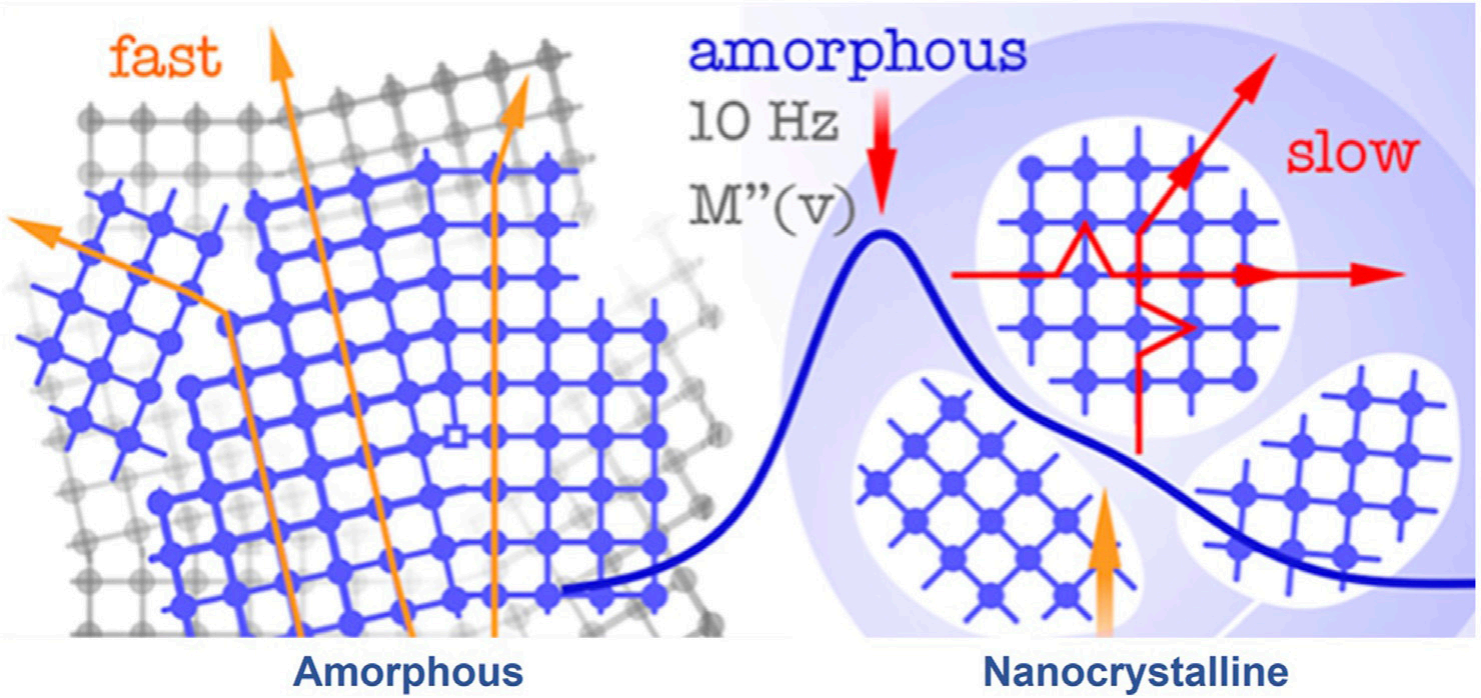
A schematic of a SPE with ion transport mechanism. Reproduced with permission from (Schweiger et al., 2022) with permission from the Royal Society of Chemistry.

SPEs typically comprise both crystalline and amorphous phases, forming when an inorganic salt is coordinated within the polymer matrix. In this process, low lattice energy salts dissolve in a polar polymer matrix, distinguishing them from other ionic conductors. This differentiation is crucial because it has been found that cations play a pivotal role in direct current (DC) ionic conductivity. According to a cationic transport theory in high molar mass polymer electrolytes, long-range cation transport primarily occurs through dissociative steps, involving coordination, de-coordination, and re-coordination along the polymer chain. This allows cations to move between nearby coordinating sites on the host molecule or onto another host molecule, as illustrated in Figure 2.

Thus, it can be deduced that the DC ionic conductivity of a SPE may not experience significant enhancement through the formation of non-labile bonds with the polar groups of the host polymer. Recent review articles propose that polymer chains fold into cylindrical tunnels where the functional groups coordinate with cations. These cylindrical tunnels then form channels that facilitate cation movement. Consequently, ion transport is primarily observed in the amorphous regions of the polymer. However, it is essential to highlight that the activity of the salt also plays a crucial role in governing charge transport within the electrolyte. It influences the potential between the two phases, ultimately impacting the overall electrochemical performance of an electrochemical cell system.

In this context, epoxy-based SPEs are formulated by combining a durable, cross-linkable DGEBA epoxy resin with lithium-solvating oligoethers (i.e., tetraethylene glycol dimethyl ether, TEGDME), weak-binding lithium salts (i.e., LiTFSI), and highly conductive ionic liquids (i.e., 1-butyl-3-methylimidazolium bis(trifluoromethylsulfonyl)imide, BMIM-TFSI). This combination is achieved through a straightforward, one-pot epoxy ring-opening polymerization process. Typically, the presence of dissolved lithium salts in epoxy-based SPEs can impose restrictions on ion-conducting properties. Consequently, to enhance the multifunctional properties, including ionic conductivity, mechanical strength, and thermal stability of these SPEs, various additives have been introduced. These additives encompass inorganic ceramic nanomaterials (Al_2_O_3_, SiO_2_, MgO, TiO_2_), ethylene oxides (EO), lithium sulfides (Li_10_GeP_2_S_12_ and Li_7_P_3_S_11_), lithium hydrides (LiBH_4_, Li_3_AlH_6_, Li_2_BH_4_NH_2_), and lithium halides (Li_1.8_N_0.4_Cl_0.6_, Li_2_CdCl_4_, Li_3_YCl_6_, Li_3_InBr_3_Cl_3_) (Zheng et al., 2020).

### 2.2 Classification

Polymer-based electrolytes have emerged as promising materials in the research and development of solid-state electrochemical devices. This is primarily attributed to their characteristics of high ionic conductivity, excellent mechanical properties, and favorable interfacial stability for electrodes. Typically, these polymer hosts are thermoplastic polymers, including polyacrylonitrile (PAN), poly (ethylene oxide) (PEO), poly (vinylidene fluoride) (PVDF), and polymethyl methacrylate (PMMA). Thermoplastic polymer-based electrolytes exhibit one-dimensional linear molecular chain structures, which are less effective in inhibiting the growth of lithium dendrites (Yu et al., 2023). In this context, crosslinked thermosetting polymers such as epoxy offer high dimensional stability, helping to prevent the formation of lithium dendrites by restricting the movement of polymer chains. Various filler materials have been incorporated into the epoxy matrix to enhance the multifunctional properties of the electrolytes. Depending on the specific filler materials used, epoxy-based SPEs can be categorized into dry polymer electrolytes (DPEs), plasticized polymer electrolytes (PPEs), gel polymer electrolytes (GPEs), and composite polymer electrolytes (CPEs).

#### 2.2.1 DPEs

Epoxy-based DPEs involve the dissolution of inorganic salts within a functional (polar) polymer matrix to create an ion-conducting solid-state electrolyte. In this process, electrostatic forces play a critical role in the interactions between metal ions and the polar groups of the polymer, leading to the formation of coordinating bonds along the polymer chains (Rivas et al., 2003). The effectiveness of these interactions is influenced by various factors, including the nature of the functional groups attached to the polymer backbone, the composition and spacing between functional groups, molecular weight, degree of crosslinking, the nature and charge of the metal cation, and the presence of counter ions. When subjected to an electric field, the cations can move between two coordinated sites within the electrolyte, facilitating ion conduction.

#### 2.2.2 PPEs

PPEs are formulated by blending a polymer host with low molecular weight plasticizers, such as ethylene carbonate, propylene carbonate, and PEG. The incorporation of these plasticizers into the electrolyte increases the amorphous portion of the hybrid polymer electrolyte and decreases the crystallinity of the host polymer. As a result, the glass transition temperature of the PPE systems decreases, leading to an improved charge transfer rate. Additionally, the inclusion of low molecular weight plasticizers enhances the salt dissociation capability within the composite systems. However, the introduction of these plasticizers has the effect of reducing the number of active centers and weakening the intermolecular and intramolecular forces between the polymer chains (Prabakaran et al., 2017). Consequently, this reduction results in a decrease in the mechanical and thermo-mechanical properties of PPEs and reduces the structural rigidity of the three-dimensional structure formed during the drying process.

#### 2.2.3 GPEs

GPEs have gained significant attention in the field of polymer electrolytes because of their ability to combine the advantages of both solid-state and liquid-type electrolytes (Schutzeichel et al., 2019). This combination offers benefits such as enhanced safety and high ionic conductivity. The preparation of epoxy-based GPEs involves blending different plasticized organic polymer electrolytes with suitable liquid solvents into epoxy resin characterized by a high dielectric constant and low viscosity. The introduction of these liquid solvents into the epoxy resin leads to an increase in both the number and size of amorphous regions owing to liquid adsorption (Zhang et al., 2022). Consequently, the ion conducting rate within the electrolytes is enhanced through the interconnected network of amorphous regions, resulting in an improved charge transfer rate and higher ionic conductivity of the GPEs. However, it is worth noting that while GPEs can achieve high ambient conductivities, they still share some drawbacks with PPEs, including issues related to volatile release and increased reactivity with the metal electrode.

#### 2.2.4 CPEs

The concept of epoxy-based CPEs primarily aims to enhance the multifunctional properties of structural polymer electrolytes. These CPEs incorporate various inorganic materials, carbonaceous substances, and different polymers, resulting in improved overall mechanical, electrical, and thermal performance. By carefully controlling the type and quantity of incorporated filler materials, the dielectric permittivity of the CPEs can be adjusted to suit specific needs. For example, the combination of ceramic fillers and polymer materials can yield new hybrid composite materials with high dielectric constants. When these inorganic fillers are in nano-dimension, the resulting composites are referred to as nanocomposite polymer electrolytes (Yang et al., 2022). The shape, size, morphology, and volume fraction of the added filler particles play a crucial role in determining both the mechanical strength and ionic conductivity of the CPEs.

### 2.3 Preparation techniques

#### 2.3.1 Solution casting

**FIGURE 3:**
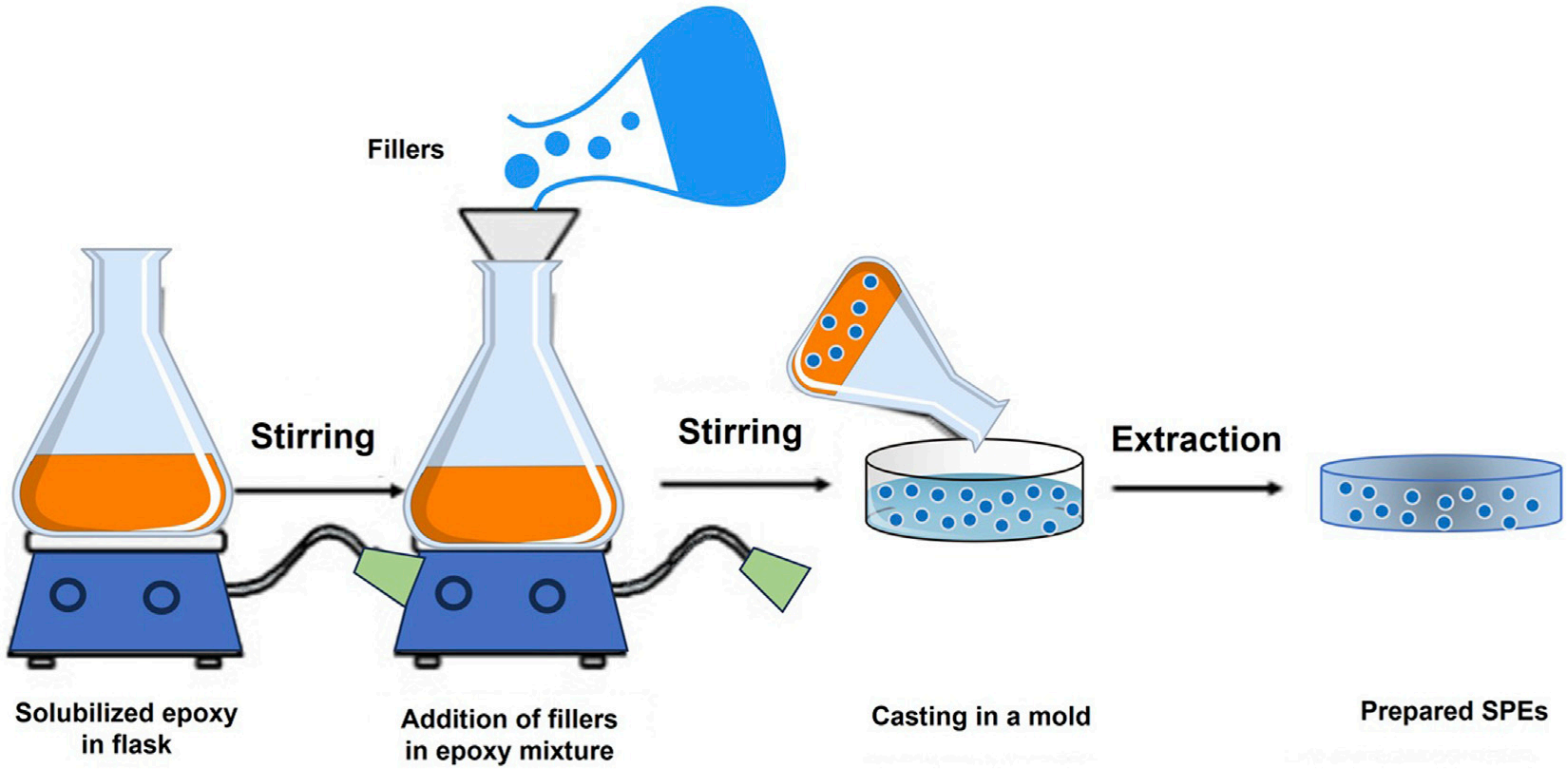
Schematic diagram of the solution casting process for SPEs.

The fabrication process for epoxy-based CPEs typically involves the conventional solution casting method. In this method, filler materials are blended with a solubilized epoxy resin while under continuous stirring. The resulting composite solutions are then poured into a mold and subjected to either drying or solvent evaporation to produce the final CPEs. Generally, the solubilized epoxy resin is prepared by dissolving the necessary amount of epoxy resin in a suitable solvent, such as water, alcohol, or an organic solvent (Singh et al., 2022). As depicted in Figure 3, a schematic diagram illustrates the preparation process for epoxy-based CPEs with nanofillers using the solution casting method. The procedure commences by pouring the solubilized epoxy resin into a container, stirring it, and allowing it to degas for a specified duration to release any trapped air bubbles. Subsequently, the appropriate amount of compatible hardener is added to the epoxy. Following this, the predetermined quantity of fly ash nanofillers is incorporated, either manually or with mechanical stirring while maintaining continuous agitation. The solution is further degassed to eliminate air bubbles for approximately 30 min. The prepared solution is then cast into a pre-prepared mold, coated with silicone, and left to cure according to the predetermined curing conditions and time. Finally, the casted SPE is removed from the mold and may undergo additional post-curing at a higher temperature for an additional hour.

The physical, electrical, and mechanical properties of produced SPEs are influenced by various factors. These encompass the viscosity of the epoxy resin, the uniform distribution of filler materials, taking into account their stereochemistry and shapes, the type of hardener employed, the inclusion of additives, and the prevailing environmental conditions during fabrication. Moreover, several processing parameters, such as temperature, stirring frequency, agitation, and curing rate, significantly impact the properties of the cast materials (Wendong et al., 2021). It is noteworthy that SPEs manufactured via solution casting at ambient temperatures tend to display less deterioration and degradation in comparison to composites produced through melt compression molding. This characteristic makes them a preferable choice for certain applications.

#### 2.3.2 Solvent-free casting

**FIGURE 4:**
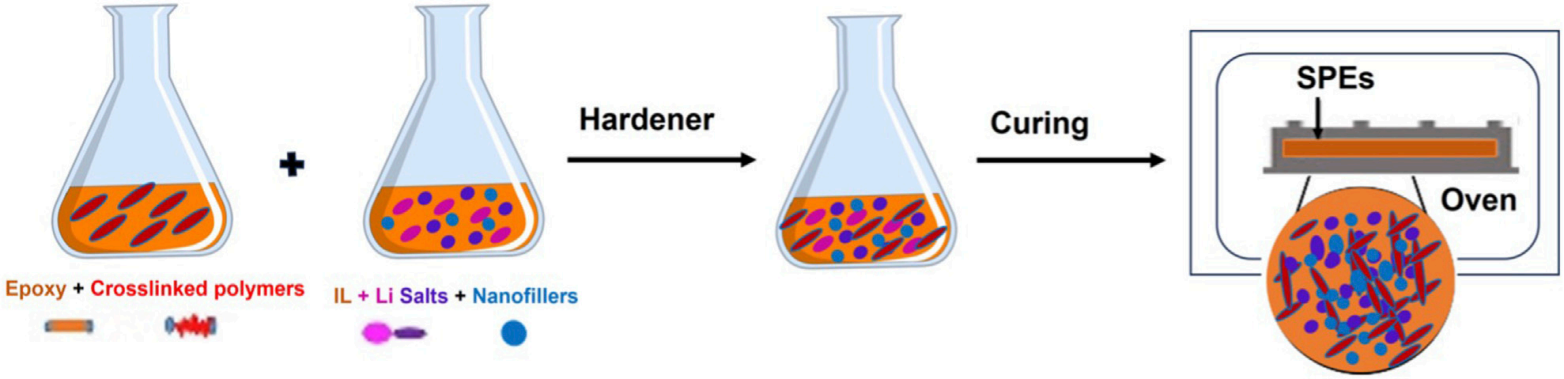
Schematic diagram of the solvent-free casting process for SPEs.

The solvent-free preparation technique for SPEs is generally simpler compared to the solution casting method. In this approach, a mixture of the epoxy resin and ionic liquid is blended and degassed at room temperature for few minutes. Following this, a stoichiometric amount of the hardener is introduced to the epoxy-ionic liquid mixture to achieve a homogeneous and viscous blend. This mixture is further stirred and degassed. Subsequently, the viscous mixture is poured into a metallic mold, previously coated with a mold release agent. The filled mold is then placed in a vacuum oven open to the air for the curing process. After finishing the curing, the solvent-free SPE samples are removed from the mold. Similarly, epoxy-based GPEs and CPEs can also be prepared using a similar approach. In these instances, before adding the hardener, lithium salts in ionic liquids, nanofillers, and crosslinked polymers are incorporated into the epoxy monomer. The process for their preparation is elucidated in Figure 4.

The properties of solvent-free polymer electrolyte films, encompassing mechanical strength, ionic conductivity, and bending flexibility, can be fine-tuned by adjusting the quantities of ionic liquids, crosslinked polymers, and nanofillers used in the SPEs. This adaptability facilitates the customization of electrolyte films to meet specific requirements. The preparation of epoxy-based GPEs is particularly straightforward using this method. Gelled polymer electrolyte networks can be easily formed by manipulating the amounts of crosslinked plasticizers at room temperature and controlling the curing time (Muñoz et al., 2021). The plasticizer is adjusted based on the combined weight of each gelled polymer electrolyte network, comprising the liquid plasticizer and the solvent-free polymer electrolyte network. This approach offers a convenient means of tailoring the properties of GPEs to align with desired specifications.

### 2.4 Multifunctional properties

#### 2.4.1 Ionic conductivity

Ionic conductivity in an electrolyte quantifies the ability of ions to conduct electricity when subjected to an electric field. The Arrhenius or Vogel-Tammann-Fulcher (VTF) model is a prevalent approach used to depict the ionic conductivity of an electrolyte. The ionic conductivity (*σ*) of an electrolyte can be mathematically expressed utilizing Arrhenius’ equation (Chen et al., 2016):

$$\sigma =\sum_{i}q_{i}c_{i}\mu_{i}=A\;\exp\left(-\frac{E_{a}}{RT}\right)$$ (1)

Here, *q*_i_ signifies the charge number of ions, *c*_i_ denotes the charge concentration, *μ*_i_ represents the mobility of the charged species, *E*_a_ is the activation energy, *A* stands for the Arrhenius constant, *R* is the molar gas constant, and *T* indicates the temperature. The *σ* of epoxy-based polymer electrolytes is evaluated using electrochemical impedance spectroscopy (EIS) (Su et al., 2022) and is calculated by

$$\sigma =\frac{L}{R_{b}\times S}$$ (2)

where *L* represents the thickness of the epoxy electrolyte, *R*_b_ is the bulk resistance, and *S* denotes the contact area between the electrode and the electrolyte.

The activation energy for the ionic conduction of epoxy electrolyte can be determined from Eq. 1, and the value of *σ* obtained from Eq. 2. The ionic conductivity of an epoxy electrolyte increases with an increase in the molar ratio of lithium salt to etheric oxygen in the electrolyte. However, a higher concentration of lithium salt may limit the ionic conductivity of the system as it restricts the movement of polymer chains, which form the ion transport paths. Sometimes, the addition of nano-fillers in the epoxy resin enhances the ionic conductivity by increasing the amorphous content in the composite system. These filler materials act as intercalating agents in the epoxy resin, leading to less regular arrangement of epoxy polymer segments. This enhanced chain segment motion helps increase the ionic conductivity of the electrolyte system.

The activation energy for the ionic conduction of epoxy electrolyte can be determined using Eq. 1 with the value of *σ* obtained from Eq. 2. Notably, the ionic conductivity of an epoxy electrolyte exhibits an upward trend with an increase in the molar ratio of lithium salt to etheric oxygen within the electrolyte. Nevertheless, a higher concentration of lithium salt may impose limitations on the ionic conductivity by constraining the movement of polymer chains, crucial for forming ion transport paths. Introducing nano-fillers into the epoxy resin can enhance ionic conductivity by augmenting the amorphous content in the composite system. These filler materials serve as intercalating agents in the epoxy resin, leading to a less regular arrangement of epoxy polymer segments. This heightened chain segment motion contributes to an increased ionic conductivity within the electrolyte system.

The quantity of transferred ions in an epoxy electrolyte-based symmetric battery system is determined utilizing a steady-state current method, (Su et al., 2022), and is given by

$$t_{+}=\frac{I_{s}\left(∆V-I_{0}R_{0}^{\text{ct}}\right)}{I_{0}\left(∆V-I_{s}R_{s}^{\text{ct}}\right)}$$ (3)

where *I*_0_ and *I*_s_ represent the initial and steady-state current values, $R_{0}^{\text{ct}}$ and $R_{s}^{\text{ct}}$ denote the electrolyte/electrode interface resistance measured before and after polarization, and ∆*V* is the potential difference. The ion transport in SPEs occurs through intra- or inter-chain hopping, and this phenomenon is contingent on the segmental movement of polymer chains. Considering the movement of polymer chains in an amorphous SPE, the VTF model is the prevailing theory employed to elucidate the ionic conductivity of SPEs (Bobade et al., 2009), and *σ* can be expressed as

$$\sigma =\frac{\sigma_{0}}{\sqrt{T}}\exp\left(-\frac{B}{T-T_{0}}\right)$$ (4)

where *T*_0_ is the reference temperature, usually positioned between 10 and 50 K below the glass transition temperature (*T*_g_), and *B* is the pseudo activation energy represented in a unit of *E*_a_/*R*. Therefore, considering the distinctive compositions and architectures of epoxy-based SPEs, a combination of both the Arrhenius and VTF equations is employed to determine their total conductivity.

#### 2.4.2 Electrochemical stability

Indeed, a meticulous design of SPEs is essential, focusing on key attributes including high ionic conductivity, low electronic conductivity, and the capability to preserve chemical and electrochemical stability at the cathode and anode interfaces throughout cycling. Evaluating the electrochemical stability window of epoxy-based composite electrolytes across the voltage range of 0–5 V is crucial for this purpose, and it is accomplished using linear sweep voltammetry (LSV) with a specific scan rate (Yao et al., 2019). The electrochemical stability window of an SPE denotes the working electric potential range wherein the SPE undergoes neither oxidation nor reduction. LSV tests typically use a three-electrode setup, with lithium metal as the reference electrode, stainless steel as the counter electrode, and the epoxy electrolyte film serving as a separator between the lithium metal and stainless steel. It has been demonstrated that composite electrolytes exhibit higher electrochemical stability when compared to pure epoxy electrolytes. An effective strategy for enhancing the working voltage of epoxy-based SPEs involves the incorporation of conductive nanofillers like graphene, carbon nanotubes, silicon dioxide, and others into the electrolytes. However, the incorporation of these filler materials should be carefully controlled, considering factors such as monitoring ion transport mechanisms through the polymer chains, maintaining filler size, concentration, and hybridization strategies. This enhancement in electrochemical stability is pivotal for the performance and safety of electrochemical devices utilizing these electrolytes.

#### 2.4.3 Mechanical strength and flexibility

In structural applications, epoxy-based SPEs must satisfy the primary requirement of possessing high mechanical strength and stability without compromising their ionic conductivity. Mechanical strength is crucial for prolonged cycling, high energy density, and high voltage application, as it ensures the material’s ability to withstand physical forces. However, the addition of lithium salts, ionic liquids, and crosslinked low molecular weight polymers typically results in a decrease in the mechanical robustness of epoxy-based SPEs. To tackle this challenge, various high-strength carbonaceous and ceramic nanofillers are incorporated to enhance the mechanical properties of epoxy electrolytes.

For instance, Li et al. (2018) demonstrated that incorporating 6 wt% TiO_2_ nanofillers in mesoporous TiO_2_-doped epoxy-based composite electrolytes increased the compression strength from 3.91 MPa to 5.06 MPa. In another study, Kwon et al. (2018) developed a multifunctional SPE with a high Young’s modulus (approximately 1 GPa), double that of pure epoxy, by introducing 5 vol% of Al_2_O_3_ into the electrolyte. Nevertheless, it is noteworthy that excessive nanofiller addition in SPEs can decrease the mechanical properties of the electrolytes because of agglomeration phenomena. Hence, careful optimization of nanofiller content is essential to achieve the desired balance between mechanical strength and ionic conductivity in epoxy-based SPEs.

#### 2.4.4 Thermal stability

Thermal stability is a crucial property for epoxy-based electrolytes to ensure their applicability in practical scenarios. A SPE is deemed thermally stable if its decomposition rate is negligible under elevated temperatures. The evaluation of thermal stability typically involves measuring weight reduction as a function of temperature through thermogravimetric analysis at varying heating rates in an inert atmosphere. Researchers have noted a slight weight loss at lower temperatures, attributed to solvent and moisture evaporation. The most substantial weight loss typically occurs around 150 °C, indicating polymer matrix breakdown (Shirshova et al., 2022). In the case of epoxy-based composite electrolytes, the decomposition usually initiates at a higher temperature. This improved thermal stability results from the incorporation of various nanofillers or solid acid compounds, enhancing the ability of SPEs to withstand high temperatures. These fillers act as protective barriers, reducing heat transfer and preventing the release of harmful byproducts. For instance, Qing et al. (2015) developed a cesium dihydrogen phosphate-incorporated epoxy composite electrolyte, exhibiting stable ionic conductivity up to 259 °C under a 30% H_2_O/Ar atmosphere for 50 h. This heightened thermal stability renders epoxy-based composite electrolytes suitable for applications where exposure to high temperatures is anticipated.

## 3 Potential application of epoxy-based SPEs

### 3.1 Supercapacitors

**FIGURE 5:**
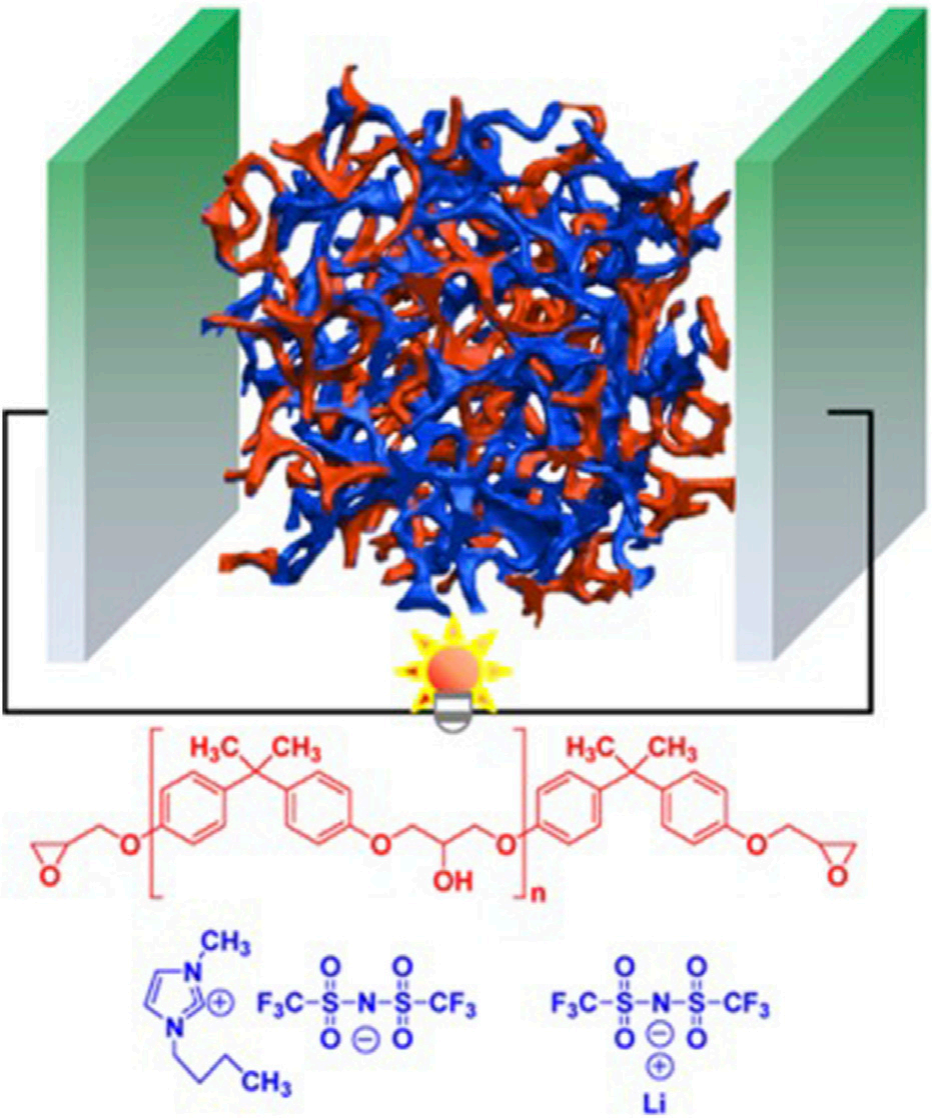
Schematic diagram of the epoxy/SPE-based structural supercapacitors. Reproduced with permission from (Kwon et al., 2018) with permission from the American Chemical Society.

Epoxy-based SPEs show significant promise in advancing multifunctional structural supercapacitors, configured as sandwich structures comprising two electrodes connected through an epoxy-based solid electrolyte, as illustrated in Figure 5. The fabrication of these sandwich structures can be accomplished through processes like compression molding at specific pressures or resin transfer molding (Lingappan et al., 2022). The manufacturing process typically involves following key steps.

(i) Electrode preparation: mesoporous or nanoporous electrodes are created, and their surface morphology is modified to enhance conductivity and establish an effective charge storage interface, utilizing various electrode materials such as silicon, carbon fiber, or carbon paper.
(ii) Electrolyte preparation: epoxy-based electrolytes are produced using different techniques as described in Section 2.3.
(iii) Curing: the casted sandwich structures are allowed to cure at room temperature or a specific elevated temperature, depending on the cure kinetics of the resins and hardeners used.
(iv) Practical application: the resulting sandwich structures function as multifunctional structural supercapacitors for various real-world applications.

These epoxy-based structural supercapacitors exhibit a range of multifunctional properties detailed in Table 1, making them suitable for applications where high mechanical strength, ionic conductivity, and energy storage capabilities are crucial.

**TABLE 1:** List of epoxy/SPE-based multifunctional structural supercapacitors.^a^

Configuration			Properties		Reference
Electrode	Electrolyte	Separator	Capacitance (F g^–1^)	Strength (MPa)	
WCF	Epoxy/CF_3_SO_3_Li	GF weave	10^−8^	42	Snyder et al. (2007b)
ACF	TEABF_4_/PC/Epoxy	Cellulose	0.007	36.7	Reece et al. (2019)
CF	PVDF/LiTf/Epoxy	GF weave	0.0116	47.5	Xu et al. (2021)
WCF/MWCNTs	Epoxy/LiTf	GF weave	0.125	21.3	Hudak et al. (2017)
WCF/MnO_2_/Silane	Epoxy/BMIMTFSI	GF weave	10.5	103	Huang et al. (2022)
WCF/CNT	Epoxy/EMIMTFSI/LiTFSI	GF weave	0.01	153	Asp and Greenhalgh (2014)
WCF	Epoxy/EMIMTFSI/LiTFSI	GF weave	0.007	292	

^a^ WCF (plain woven carbon fiber), GF (glass fiber), ACF (activated carbon fiber), TEABF_4_ (tetraethylammonium tetrafluoroborate), PC (polycarbonate), CF (carbon fiber), PVDF (polyvinylidene fluoride), LiTf (lithium trifluoromethanesulfonate), MWCNT (multiwalled carbon nanotubes), BMIMTFSI (1-butyl-3-methylimidazolium bis(trifluoromethylsulfonyl)imide), EMIMTFSI (1-ethyl-3-methylimidazolium bis(trifluoromethylsulfonyl)imide, LiTFSI (lithium bis(trifluoromethanesulfonyl)imide).

### 3.2 Batteries

**FIGURE 6:**
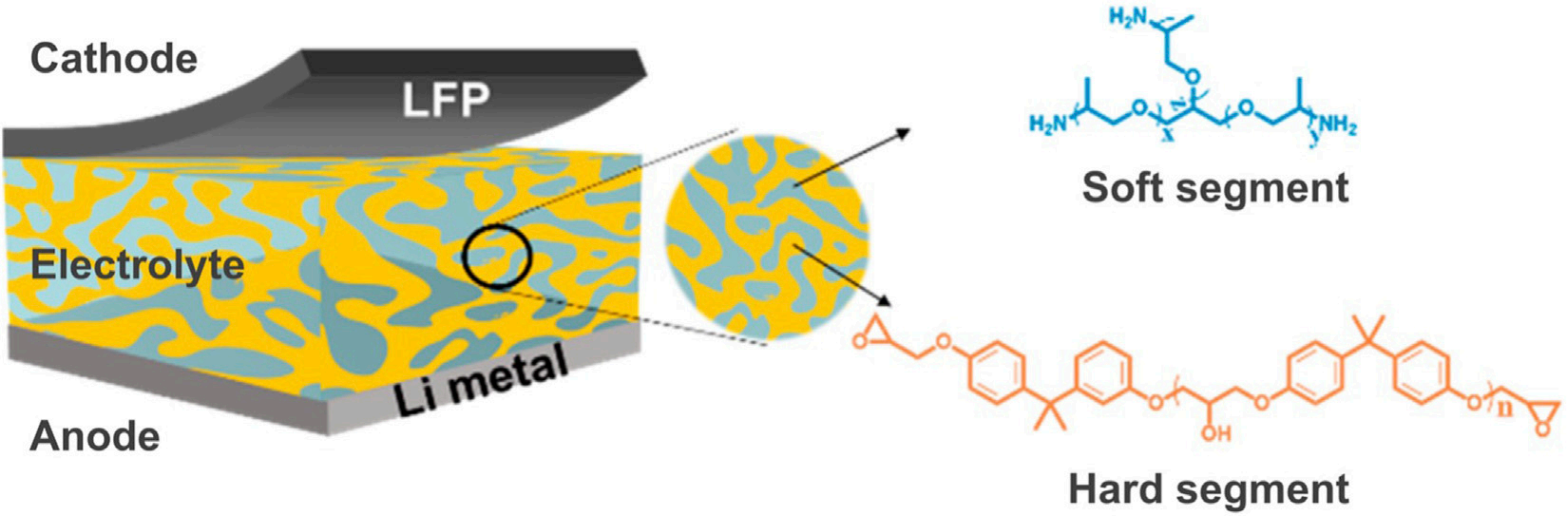
Schematic diagram of the epoxy/SPE-based structural batteries. Reproduced with permission from (Zeng et al., 2021) from the American Chemical Society.

Epoxy-based solid-state batteries are gaining prominence in the field of energy storage because of their non-flammable nature, design flexibility, and leakproof properties in practical applications. Historically, there were no solid-state materials capable of enabling ion mobility and generating a sufficient electric current within the battery (Asp et al., 2019). However, the discovery of epoxy-based multifunctional electrolytes has significantly propelled the development of solid-state structural batteries. These batteries share a mechanism akin to lithium-ion batteries, but they serve the dual purpose of acting as both a mechanical load bearer and an energy storage device simultaneously. Typically, an epoxy-based solid-state battery comprises an anode, cathode, and a composite solid-state electrolyte (Figure 6). In this design, the electrolyte serves as both an ionic conductor and a separator, eliminating the need for a conventional separator. Composite solid-state electrolytes can be categorized into four primary groups, including the incorporation of inorganic fillers within the epoxy matrix, heterogeneous layered structures, three-dimensional inorganic continuous frameworks with polymer infiltration, and open-framework-related composite electrolytes.

The electrochemical reactions in epoxy-based solid-state batteries closely mimic those found in conventional lithium-ion batteries with liquid electrolytes during the electrochemical process. When charging, lithium ions travel through the composite solid-state electrolyte to the anode, typically composed of lithium foil, while the generated electrons move from the cathode to the anode through the external circuit. Conversely, during discharging, these processes are reversed. It is noteworthy that the transport of lithium ions in epoxy-based composite solid-state electrolytes is more intricate compared to typical liquid electrolytes. The multifunctional properties of several epoxy-based solid-state batteries are outlined in Table 2.

**TABLE 2:** List of epoxy/SPE-based multifunctional structural batteries.^b^

Cell configuration	Properties				Reference
	Ionic conductivity (S m^–1^)	Strength (MPa)	Modulus (GPa)	Thermal stability (°C)	
(LiCoO_2_/GNP)|[LiClO_4_/PC(80):DGEBA)(20)] with 20 wt% LiTFSI/PC|CF	81 × 10^−6^	82.1 ± 0.37	1135 ± 23.1	-	Javaid and Ali (2018)
(LiCoO_2_/GNP)|[LiClO_4_/PC(60):DGEBA) (40)] with 40 wt% LiTFSI/PC|CF	11.9 × 10^−4^	53.1 ± 0.82	221 ± 10.1	-	
(LiCoO_2_/GNP)|[LiClO_4_/PC(40):DGEBA (60)] with 60 wt% LiTFSI/PC|CF	138 × 10^−4^	4.58 ± 0.11	6.19 ± 0.32	-	
(LiCoO_2_/GNP)|[LiClO_4_/PC(20):DGEBA (80)] with 80 wt% LiTFSI/PC|CF	158 × 10^−3^	0.23 ± 0.01	1.45 ± 0.03	-	
LFP|LiTFSI in PEO/PGA| Li	5.61 × 10^−2^	-	10.5	<80	Yu et al. (2021)
Na_3_V_2_(PO_4_)_3_|epoxy-NASICON|Na	1.45 × 10^−2^	35 ± 5	0.01	∼400	Lim et al. (2020)

^b^ LiCoO_2_ (lithium cobalt oxide), GNP (graphene nanoplatelets), LiClO_4_ (Lithium bis(trifluoromethanesulfonyl)imide), PC (propylene carbonate), GR (graphite), EMIMBF_4_ (1-Ethyl-3-methylimidazolium tetrafluoroborate), LFP (lithium iron phosphate), Na_3_V_2_(PO_4_)_3_ (sodium vanadium phosphate), NASICON (sodium super ionic conductor), NPS (sulphur nanoparticles), SP (conductive carbon Super P), ER (bisphenol A diglycidyl ether), PEI (polyethylenimine), DOL (1,3-dioxolane), DME (1,2-dimethoxyethane).

## 4 Challenges and perspectives

### 4.1 Understanding of ion conduction mechanisms

Many physical models have been developed to understand intrinsic ion transport in pure solid-state electrolytes (SSEs), but the majority of these models are centered on the intrinsic ion conduction mechanisms within SSEs. Therefore, several contentious theories still need clarification, especially for composite solid electrolytes (CSEs). The ionic conduction mechanisms in CSEs are influenced by different material compositions and phases, especially in the complex interfacial regions between inorganic fillers/polymers, polymers/polymers, and CSEs/electrodes. These complexities have a significant impact on lithium-ion conduction in various CSE systems. Therefore, enhancing the understanding of the intricate ion conduction pathways in epoxy-based CSEs is crucial. Achieving this understanding requires a combination of theoretical and experimental techniques to investigate lithium-ion transport behaviors in epoxy-based CSEs, including their thermodynamic and kinetic behavior during the migration process.

Various physical models have been developed to comprehend intrinsic ion transport in pure solid-state electrolytes (SSEs). However, the majority of these models are focused on understanding the intrinsic ion conduction mechanisms within SSEs. Consequently, several contentious theories remain to be clarified, particularly concerning SPEs. In SPEs, the ionic conduction mechanisms are influenced by diverse material compositions and phases, particularly in the intricate interfacial regions between inorganic fillers/polymers, polymers/polymers, and SPEs/electrodes. These complexities significantly impact lithium-ion conduction in different SPE systems. Thus, it becomes crucial to enhance our understanding of the intricate ion conduction pathways in epoxy-based SPEs. Achieving this understanding necessitates a combination of theoretical and experimental techniques to investigate lithium-ion transport behaviors in epoxy-based SPEs, encompassing their thermodynamic and kinetic behavior during the migration process.

### 4.2 Enhancement of conductivities

Despite recent advancements in epoxy-based SPE conductivities, the prevailing majority of sources still report current conductivities within the range of 10^−4^ to 10^−5^ S cm^–1^ at room temperature. Only a limited number of studies have managed to attain conductivities exceeding 10^−3^ S cm^–1^. To fully unlock the potential of solid-state batteries and expedite their practical integration, achieving conductivities within the range of 10^−3^ to 10^−2^ S cm^–1^ at ambient temperatures is imperative, making them comparable to liquid electrolytes. This level of conductivity is vital for the widespread adoption of solid-state batteries across diverse applications.

### 4.3 Optimization of electrochemical stabilities

The sustained performance of epoxy-based SPEs hinges on the stability of interactions between the electrolytes and electrodes, spanning chemical, electrochemical, thermal, and mechanical aspects. These stability factors collectively govern the performance of the electrolytes over an extended period. However, it is important to acknowledge that challenges related to stability can arise in epoxy-based CPEs because of the incorporation of inorganic fillers, the formation of double or sandwiched layered structures, and three-dimensional inorganic continuous frameworks with polymer infiltration. Consequently, optimizing the stability of epoxy-based CPEs is crucial to enhance their long-term performance and energy storage capabilities.

### 4.4 Economic and technological feasibilities

The production costs associated with epoxy-based SPEs, particularly those featuring intricate structural designs and components, often surpass those of conventional liquid electrolytes used in lithium batteries. Consequently, it becomes imperative to focus on the design and manufacturing of SPEs with an emphasis on enhancing their economic viability. This can entail various strategies, such as opting for more cost-effective components or those containing fewer rare Earth elements, refining synthetic processes to conserve energy and raw materials, and developing SPEs with composite structures that are both practical and cost-efficient. These considerations play a vital role in establishing solid-state batteries as a competitive and sustainable choice in the energy storage industry.

## 5 Conclusion

This review offers an overview of epoxy resin-based SPEs and their potential applications. It delves into the fundamental composition of epoxy-based polymer electrolytes and elucidates their ion transport mechanisms. The development of various categories of SPEs, including PPEs, GPEs, CPEs, and DSPEs, is explored, shedding light on the constituent materials and the incorporation of nanofillers. The preparation methods for these polymer electrolytes, such as solvent casting and solvent-free casting, are meticulously detailed. The multifunctional properties of epoxy-based SPEs, encompassing electrochemical, mechanical, and thermal characteristics, are thoroughly explained. Recognizing the inadequacy of the Arrhenius and VTF models alone to describe ion transport mechanisms in these materials, a combination of both models is proposed for assessing the total conductivity of epoxy-based SPEs. The hindrance posed by crystalline phases in these electrolytes to ion transport through the polymer network, limiting DC ionic conductivity, is identified. To overcome this limitation, the development of new polar polymers with higher amorphous content is advocated.

The article also emphasizes the role of nanofiller-reinforced epoxy-based composite polymer electrolytes in enhancing amorphous content and consequently boosting ionic conductivity. These composite electrolytes not only improve DC ionic conductivity but also enhance electrochemical stability, mechanical strength, and thermal stability. The potential applications of epoxy-based SPEs in multifunctional energy storage devices, such as supercapacitors and batteries, are discussed. However, the chapter acknowledges that certain technical challenges, including understanding ion conduction mechanisms in CSEs, optimizing electrochemical stabilities, and addressing economic and technological feasibility issues, need further exploration. Researchers are encouraged to continue developing epoxy-based solid polymer electrolytes and energy storage devices.

The article underscores the significance of nanofiller-reinforced epoxy-based SPEs in augmenting amorphous content, consequently elevating ionic conductivity. These composite electrolytes not only enhance DC ionic conductivity but also improve electrochemical stability, mechanical strength, and thermal stability. The potential applications of epoxy-based SPEs in multifunctional energy storage devices, such as supercapacitors and batteries, are thoroughly examined. However, the chapter acknowledges certain technical challenges, including comprehending ion conduction mechanisms in SPEs, optimizing electrochemical stabilities, and addressing economic and technological feasibility issues, which necessitate further exploration. Researchers are encouraged to persist in the development of epoxy-based SPEs and energy storage devices.
